# A novel IgE antibody targeting the prostate-specific antigen as a potential prostate cancer therapy

**DOI:** 10.1186/1471-2407-13-195

**Published:** 2013-04-17

**Authors:** Tracy R Daniels-Wells, Gustavo Helguera, Richard K Leuchter, Rafaela Quintero, Maggie Kozman, José A Rodríguez, Elizabeth Ortiz-Sánchez, Otoniel Martínez-Maza, Birgit C Schultes, Christopher F Nicodemus, Manuel L Penichet

**Affiliations:** 1 Division of Surgical Oncology, Department of Surgery, David Geffen School of Medicine, University of California, Los Angeles, CA, USA; 2 The Molecular Biology Institute, University of California, Los Angeles, CA, USA; 3 Department of Microbiology, Immunology, and Molecular Genetics, David Geffen School of Medicine, University of California, Los Angeles, CA, USA; 4 Jonsson Comprehensive Cancer Center, University of California, Los Angeles, CA, USA; 5 Department of Obstetrics and Gynecology, David Geffen School of Medicine, University of California, Los Angeles, CA, USA; 6 Department of Epidemiology, Fielding School of Public Health, University of California, Los Angeles, CA, USA; 7 Advanced Immune Therapeutics, Inc, Charlestown, MA, USA; 8 Current Affiliation: Momenta Pharmaceuticals, Inc, Cambridge, MA, USA; 9 Current Affiliation: School of Pharmacy and Biochemistry, University of Buenos Aires, Buenos Aires, Argentina; 10 Current Affiliation: Unit of Biomedical Research in Cancer, Basic Research Division, National Institute of Cancerology, Mexico City, Mexico; 11 Current Affiliation: AIT Strategies, Franconia, NH, USA

**Keywords:** Prostate-specific antigen, AllergoOncology, IgE, Hypersenstivity, Recombinant antibody

## Abstract

**Background:**

Prostate cancer (PCa) is the second leading cause of cancer deaths in men in the United States. The prostate-specific antigen (PSA), often found at high levels in the serum of PCa patients, has been used as a marker for PCa detection and as a target of immunotherapy. The murine IgG1 monoclonal antibody AR47.47, specific for human PSA, has been shown to enhance antigen presentation by human dendritic cells and induce both CD4 and CD8 T-cell activation when complexed with PSA. In this study, we explored the properties of a novel mouse/human chimeric anti-PSA IgE containing the variable regions of AR47.47 as a potential therapy for PCa. Our goal was to take advantage of the unique properties of IgE in order to trigger immune activation against PCa.

**Methods:**

Binding characteristics of the antibody were determined by ELISA and flow cytometry. *In vitro* degranulation was determined by the release of β-hexosaminidase from effector cells. *In vivo* degranulation was monitored in human FcεRIα transgenic mice using the passive cutaneous anaphylaxis assay. These mice were also used for a vaccination study to determine the *in vivo* anti-cancer effects of this antibody. Significant differences in survival were determined using the Log Rank test. *In vitro* T-cell activation was studied using human dendritic cells and autologous T cells.

**Results:**

The anti-PSA IgE, expressed in murine myeloma cells, is properly assembled and secreted, and binds the antigen and FcεRI. In addition, this antibody is capable of triggering effector cell degranulation *in vitro* and *in vivo* when artificially cross-linked, but not in the presence of the natural soluble antigen, suggesting that such an interaction will not trigger systemic anaphylaxis. Importantly, the anti-PSA IgE combined with PSA also triggers immune activation *in vitro* and *in vivo* and significantly prolongs the survival of human FcεRIα transgenic mice challenged with PSA-expressing tumors in a prophylactic vaccination setting.

**Conclusions:**

The anti-PSA IgE exhibits the expected biological properties and is capable of triggering immune activation and anti-tumor protection. Further studies on this antibody as a potential PCa therapy are warranted.

## Background

Prostate cancer (PCa) is the most frequently diagnosed cancer and the second leading cause of cancer deaths among men in the USA, with an estimated 241,740 new cases and 28,170 deaths in 2012 [1]. At its early stages, localized PCa can be curable in many cases by treatments such as surgery or radiation [2]. PCa that relapses or is found to be metastatic at diagnosis may be treated with androgen deprivation therapy [3,4]. However, despite this treatment these tumors eventually progress and become androgen-refractory within a few years [3]. Since 2004, the first line treatment of advanced castration-resistant PCa has been docetaxel, a microtubule stabilizing taxane, combined with prednisone [4,5]. However, this combination treatment strategy demonstrates a modest improvement in overall survival and less than 20% of these patients attain a 3-year survival rate [4,5]. Therefore, additional treatment options are needed, especially at the advanced or metastatic stage.

An alternative strategy to the above-mentioned therapeutic approaches is immunotherapy. PCa is attractive as a target for immunotherapy for multiple reasons: 1) the presence of organ-specific tumor-associated antigens, 2) the initial slow-growing nature of the disease allowing for adequate time for an anti-immune response to develop, and 3) immunotherapy is relatively safe given the dispensable nature of the organ [5,6]. This suggestion is supported by the fact that an immunotherapeutic strategy (sipuleucel T) was approved by the FDA in 2010 for the treatment of metastatic, castration-resistant PCa [7]. Sipuleucel-T is an autologus vaccination strategy in which peripheral blood mononuclear cells, including antigen presenting cells (APC) are isolated from the patient and are stimulated with a fusion protein consisting of prostatic acid phosphatase and the immunostimulatory cytokine granulocyte-macrophage colony-stimulating factor (GM-CSF). These cells are then reinfused back into the patient. This vaccination strategy has shown a survival advantage over docetaxel-based treatments [4,5,7,8]. Another experimental vaccination strategy (Prostvac-VF) consisting of a recombinant vaccinia virus expressing the prostate-specific antigen (PSA) and other co-stimulatory molecules has also shown promise in clinical trials of PCa [4,5,8]. However, despite these significant advances, the continued high rate of relapse and absence of a cure for metastatic disease highlights the need for additional treatments for PCa.

PSA is a 30 kDa glycoprotein serine protease expressed by prostate epithelial cells that is normally confined to the seminal fluid [9,10]. Only very small amounts of PSA reach the circulatory system in healthy men [9,11,12]. However, due to structural changes in the cancerous prostate, PSA is often found at high concentrations in the blood of PCa patients, making it a useful diagnostic marker. Different forms of PSA can be found in the blood, including free PSA or PSA complexed to other proteins. Free PSA accounts for approximately 15-20% of total PSA. In the blood, PSA can form complexes with the protease inhibitors α1-antichymotrypsin (ACT), α1-protease inhibitor (API), and α2-macroglobulin (A2M). The monomeric ACT complex, composed of 1 PSA molecule covalently linked to 1 ACT, is by far the most prevalent, comprising nearly 80% of total PSA compared to 1-2% for the A2M and API complexes [9-12]. PSA is a promising target for PCa immunotherapy because of its organ specific production. In fact, a murine bispecific antibody against human CD3 and human PSA has been shown to mediate anti-tumor effects against prostate carcinoma cells *in vitro* and *in vivo*[13]. Additionally, a rabbit IgG antibody targeting human PSA was shown to preferentially deliver a chemotherapeutic drug to PSA-expressing tumors in nude mice [14]. This immunoconjugate was preferentially retained within the PCa tumors and enhanced cancer cell death, results that were not observed in PCa tumors that did not express PSA [14]. Furthermore, the murine anti-PSA IgG1 (AR47.47) complexed with PSA showed enhanced antigen presentation by human dendritic cells (DC) and induced both CD4 and CD8 T-cell activation [15], suggesting the possibility that this antibody may interact with PSA in the blood of patients redirecting this antigen into APC for antigen processing, presentation, and T-cell activation against the tumor.

The efficacy of antibody therapies for the treatment of cancer has been demonstrated in multiple cases [16,17]. Examples include trastuzumab (Herceptin^®^, a humanized anti-HER2/*neu* IgG1) and rituximab (Rituxan^®^, a mouse/human chimeric anti-CD20 IgG1). Although most antibodies used for cancer therapy are of the IgG class [16,17], antibodies of the IgE class have various properties that may be advantageous over IgG as potential cancer therapeutics. These properties include 1) the low endogenous concentration in serum (0.02% of circulating immunoglobulins compared to 85% for IgG) that results in less competition for FcR occupancy, 2) the lack of an inhibitory FcεR, and 3) the superior affinity of IgE for its two FcεRs relative to IgG and its FcγRs [18,19]. There are two human FcεRs, the FcεRI that binds human IgE with high affinity (K_a_ = 10^10^ M^-1^) and is expressed on human basophils, mast cells, monocytes, macrophages, eosinophils, Langerhans cells, and DC, and the FcεRII (CD23) that binds IgE with lower affinity (K_a_ = 10^8^ M^-1^) and is expressed on human B cells, eosinophils, monocytes, macrophages, and DC [18,20,21]. Importantly, IgE antibodies have been successfully used in animal models as passive cancer immunotherapies and as adjuvants of cancer vaccines [22-25].

Given the relevance of PSA as a PCa antigen and the attractive properties of the IgE molecule, our main goal was to develop a mouse/human chimeric IgE antibody containing the variable regions of the murine antibody AR47.47. We now report the construction and expression of this novel antibody, as well as the evaluation of its properties, including its potential anti-cancer activity. We show its ability to bind the PSA antigen and the FcεRI, to induce effector cell degranulation when effectively cross-linked (but not in the presence of the natural soluble antigen), and when complexed to PSA to induce T-cell stimulation and anti-tumor activity *in vivo*.

## Methods

### Cell lines

Sp2/0-Ag14 murine myeloma cells were purchased from ATCC (American Type Culture Collection, Manassas, VA). The Chinese Hamster Ovary cell line (CHO-3D10) expressing the human FcεRIα subunit [26] and the RBL SX-38 rat basophil leukemic cell line expressing the full human receptor (α, β, and γ subunits) [27] were kindly provided by Dr. Jean-Pierre Kinet (Beth Israel Deaconess Medical Center, Boston, MA). Murine CT26 colorectal carcinoma cells expressing and secreting human PSA (CT26-PSA) [28] were kindly provided by Dr. John G. Frelinger (University of Rochester Medical Center, Rochester, NY). All cells were grown in IMDM media (Life Technologies Corporation, Carlsbad, CA) supplemented with 100 U/mL penicillin, 10 μg/mL streptomycin, and 10% (v/v) heat inactivated fetal bovine serum (FBS; Atlanta Biologicals, Atlanta, GA). Growth medium for CT26-PSA and RBL SX-38 cells was additionally supplemented with 1 mg/mL G418 (Life Technologies Corporation).

### Development of recombinant anti-PSA antibodies

The mouse/human chimeric anti-human PSA IgG1 and IgE were constructed using the DNA encoding the variable regions of the murine IgG1 AR47.47 antibody [15]. The V_L_ and V_H_ regions obtained by RT-PCR from the hybridoma expressing AR47.47 were cloned into pCR-BluntII-TOPO vectors (Life Technologies Corporation). The DNA encoding the variable regions was then subcloned into human κ light chain or the human ε (the classic secreted isoform) [29] or γ1 heavy chain expression vectors [30], respectively, all of which were obtained as kind gifts from Dr. Sheri L. Morrison (University of California at Los Angeles). The cloning strategy used to develop both antibodies is shown in Additional file 1: Figure S1. Both antibodies were expressed in the murine myeloma cell line Sp2/0-Ag14, expanded in roller bottles, and purified from cell culture supernatants. The anti-human PSA IgE was purified using an immunoaffinity column containing anti-human IgE (omalizumab, Xolair^®^, Genentech, Inc. San Francisco, CA) coupled to cyanogen bromide-activated Sepharose (GE Healthcare, Piscataway, NJ) as recommended by the manufacturer. The chimeric anti-human PSA IgG1 was purified using a Protein A-Sepharose 4B, Fast Flow immunoaffinity matrix (Sigma Aldrich, St. Louis, MO). A human HER2/*neu* IgE [25] was produced in the same manner alongside the anti-PSA IgE and was used as a non-PSA specific control (NS IgE). Rituximab (Rituxan^®^, a mouse/human chimeric anti-CD20 IgG1; NS IgG) was obtained from Hoffman La Roche (Indianapolis, IN). All antibodies were quantified using the BCA Protein Assay (ThermoFisher Scientific Inc., Walnut, CA).

### Antigen binding (ELISA)

Immunolon H-2B plates (ThermoFisher Scientific, Inc.) were coated with 5 μg/mL PSA or 10 μg/mL of the PSA peptides containing amino acids (aa) 136–148 or 137–172, which include the epitope recognized by the murine monoclonal antibody AR47.47 [15]. The plates were incubated at 4°C overnight, blocked in 3% BSA in PBS for 1 hour at room temperature, and incubated at 4°C overnight with various concentrations of purified anti-PSA IgE or a NS IgE. Binding of the IgE was detected using an alkaline phosphatase (AP)-conjugated anti-human κ-secondary antibody and a phosphatase substrate. Absorbance at 405 nm was read on a DTX880 Multimode Detector (Beckman Coulter, Fullerton, CA).

### Binding to FcεRI (flow cytometry)

5×10^5^ CHO-3D10 cells expressing human FcεRIα were detached from tissue culture dishes using 0.5 mM EDTA in PBS. Cells were incubated with either 1 μg NS IgG1 (negative control), NS IgE (positive control), or anti-PSA IgE in 50 μL IMDM + 10% FBS for 2 hours on ice. Samples were washed and binding to cells was detected by the addition of an anti-human κ-FITC (BD Biosciences, San Jose, CA). An anti-FcεRI phycoerythrin (PE)-conjugated antibody (eBioscience, San Diego, CA) was tested simultaneously to verify receptor expression. Cells were fixed with 2% paraformaldehyde and analyzed on a Becton Dickinson FACScan Analytic Flow Cytometer in the UCLA Jonsson Comprehensive Cancer Center and Center for AIDS Research Flow Cytometry Core Facility. Histograms were created using FCS express V3 (De Novo Software, Los Angeles, CA).

### HSA-PSA multi-epitope peptide construction and PSA proteins

ChromPure human serum albumin (HSA) was purchased from Jackson ImmunoResearch Laboratories, Inc (West Grove, PA) and cross-linked using the sulfosuccinimidyl 4-[N-maleimidomethyl]cyclohexane-1-carboxylate (Sulfo-SMCC; ThermoFisher Scientific Inc.) to a synthetic peptide containing the AR47.47 binding site. Human albumin was diluted in PBS to 2 mg/mL. Sulfo-SMCC was diluted in 50 mM sodium phosphate buffer to a final concentration of 20 mM and added (20 μL) to human albumin. The mixture was incubated for 30 minutes at room temperature. The reaction was stopped by adding 50 mM Tris, pH 7.2. The PSA peptide was then added (1.56 mg) at a 1 to 10 molar ratio. After a 30-minute incubation, excess cross-linker was removed using a Zeba spin desalting column with a molecular weight cut-off of 7DK (ThermoFisher Scientific, Inc.) equilibrated with PBS. Excess peptide was removed using a desalting column with a cut-off of 10K. The HSA-PSA preparation was aliquoted and stored at -80°C. Human PSA and the PSA-ACT complex both purified from human blood/seminal fluid were purchased from Lee Biosolutions, Inc. (St. Louis, MO).

### *In vitro* degranulation assay

RBL SX-38 cells were detached using 0.5 mM EDTA in PBS and 10^5^ cells were added to wells of a 48-well plate in 500 μL IMDM containing 10% FBS. Cells were allowed to adhere overnight and then sensitized with 1 μg/well anti-PSA IgE in assay buffer (5 mM KCl, 125 mM NaCl, 20 mM Hepes, 1.5 mM CaCl_2_, 1.5 mM MgCl_2_; pH 7.4) or in assay buffer alone as a negative control. The plate was then incubated in standard tissue culture conditions for 2 hours. Various concentrations of PSA, HSA-PSA (positive control), PSA-ACT, 10 μM calcium ionophore (positive control; Millipore, Billerica, MA), or assay buffer (negative control) were added and incubated with the RBL SX-38 cells for an additional 2 hours. Degranulation was quantified by the amount of β-hexosaminidase released into the cell supernatant. Using a fresh 96-well plate, 100 μL of the substrate [2.5 mM *p*-nitrophenyl-*N*-acetyl-β-D-glucosamine in 50 mM citrate buffer (50 mM citric acid, 50 mM tribasic sodium citrate, pH 4.5)] was added to 50 μL of supernatant for each sample. Reactions were quenched by the addition of 100 μL sodium carbonate buffer (50 mM sodium carbonate, 50 mM sodium bicarbonate, pH 10). Absorbance at 405 nm was determined using a DTX880 Multimode detector (Beckman Coulter, Fullerton, CA). β-hexosaminidase release is expressed as a percentage of total basophil content as determined by separate treatment with 1% Triton X-100 (maximum release). The percent degranulation was determined by the following equation [(experimental release – spontaneous release)/(maximum release – spontaneous release)] × 100.

### *In vivo* passive cutaneous (local) anaphylaxis (hypersensitivity) assay

All experimental protocols were approved by the UCLA Institutional Animal Care and Use Committee (IACUC). Since human IgE does not interact with murine FcεRI [20], BALB/c human FcεRIα transgenic mice were used for this assay (a kind gift from Dr. Jean-Pierre Kinet, Beth Israel Deaconess Medical Center, Boston, MA). In these animals, the human FcεRIα replaces its murine homolog and associates with murine FcεRIγ and FcεRIβ chains resulting in a chimeric FcεRI that can be activated by human IgE [31-33]. The expression of the FcεRI in these animals mimics the expression pattern found in humans with FcεRI being expressed on mast cells, eosinophils, basophils, DC, Langerhans cells, macrophages, and monocytes. It is important to note that these animals are not transgenic for human CD23, and thus only express murine CD23 (which does not bind human IgE [34]). The assay was performed essentially as previously described [25]. Mice were injected intradermally with PBS or 1 μg of anti-PSA IgE in a volume of 50 μL. After 4 hours, 25 μg anti-human κ (Sigma Aldrich), 50 μg PSA, or 50 μg HSA-PSA was injected intraveneously in 1% Evans blue in PBS. The mice were euthanized after 10 minutes. Leakage of the blue dye into the skin is due to a local IgE-induced inflammatory response. Color was quantified using the NIH Image J Software and intensity reported as the mean signal per pixel.

### *In vitro* antigen stimulation assay

This assay was performed as previously described [15,25]. Human peripheral blood mononuclear cells (PBMC) were obtained from healthy donors. Monocytes were isolated from the PBMC using the EasySep Human Monocyte Enrichment Negative Selection Kit (StemCell Technologies, British Columbia, Canada). Monocytes were 89-96% pure and were cultured for seven days in 4 ng/mL IL-4 and 0.1 ng/mL GM-CSF to differentiate them to DC, which were then loaded with 2 μg/mL PSA mixed with various amounts of either a NS IgG1 (0.5 to 2 μg/mL), anti-PSA IgG1 (0.5 to 2 μg/mL), or equimolar amounts of anti-PSA IgE (0.31-2.5 μg/mL). After loading for 4 hours, the DC were matured with 0.18 ng/mL IFN-α and 10 ng/mL TNF-α. DC were loaded with immune complexes prior to maturation to maximize antigen uptake, since mature DC favor antigen presentation over antigen uptake. Autologous T cells were isolated from PBMC using the EasySep Human T Cell Negative Selection kit (StemCell Technologies) and were > 90% pure. These T cells were added to cultures containing matured DC on day 8. T cells were incubated for 7 days and restimulated with freshly pulsed DC. T cells were stimulated (*in vitro* sensitization) for a total of 3 rounds. During the last stimulation round, cells were treated with Brefeldin A 4 hours after initiation of the third stimulation to block cytokine secretion, and T cells were harvested 18 hours later. These cells were then stained for CD3, CD8 and intracellular IFNγ. Activation of CD4 (CD3^+^/CD8^-^/IFN-γ^+^) and CD8 (CD3^+^/CD8^+^/ IFN-γ^+^) T cells was assessed by flow cytometry and analyzed using FlowJo (Tree Star, Inc).

### Vaccination study in human FcεR1α transgenic mice

Age-matched, male, human FcεRIα transgenic mice aged 7 to 13 weeks old were immunized with 4 μg PSA alone or complexed in a 1:1 molar ratio with either anti-human PSA IgG1 or anti-human IgE, injected subcutaneously in the left flank (4 mice per group). Age-matched, male controls were injected with buffer alone. Mice were boosted 2 weeks later (on day 15) in the same flank. Serum was collected from all animals on days 14 and 28. Mice were challenged on day 40 with 10^6^ CT26-PSA cells subcutaneously in the right flank. Tumor growth was monitored using a caliper and animals were euthanized when the tumors reached 1.5 cm in diameter. Survival was recorded from the day of tumor challenge. The Kaplan-Meier plot was generated using GraphPad Prism 4 (GraphPad Software Inc., La Jolla, CA). Significant differences in survival were determined using the Log Rank test in GraphPad Prism.

### Detection of murine anti-human PSA antibodies in the serum of vaccinated mice

Blood from vaccinated animals was collected on days 14 and 28 and was allowed to clot at room temperature for 1 hour. Serum was collected after centrifugation at 3000 rpm for 10 minutes and stored at -80°C. For the ELISA, Immunolon H-2B plates (ThermoFisher Scientific, Inc.) were coated with 1 μg/mL human PSA. The plates were incubated at 4°C overnight, washed 4 times in PBS, blocked in 3% BSA in PBS for 1 hour at room temperature, and washed with 0.1% Tween-20 in PBS. For detection of murine IgG1, mouse sera were serially diluted 3-fold starting at a dilution of 1:100 in 0.1% Tween-20 in PBS. Purified murine IgG1 AR47.47 (3-fold serial dilutions starting at 100 ng/mL) served as the positive control, while the negative control sera was obtained from unvaccinated animals (diluted in the same manner). The presence of murine IgG1 specific for PSA was detected using a rabbit anti-mouse IgG1 AP-conjugated antibody (Life Technologies) and a phosphatase substrate (Sigma Aldrich). Absorbance at 405 nm was read on a DTX880 Multimode Detector (Beckman Coulter). For detection of murine IgG2a, sera were serially diluted 2-fold starting at a 1:50 dilution in 0.1% Tween-20 in PBS. Negative control sera were obtained from unvaccinated animals and were prepared in the same manner. No murine IgG2a specific for PSA was available for a positive control. Murine IgG2a antibodies were detected with an AP-conjugated anti-mouse IgG2a (Life Technologies). Sera were considered positive if absorbance values were greater than 0.05 after a 2-hour incubation with substrate for IgG1 or a 1-hour incubation for IgG2a (negative control serum values ranged from 0.02-0.03). Significant increases in titers were determined using the Fisher’s Exact probability test (GraphPad Software Quick Calcs Online Calculator for Scientists).

## Results

### Binding analysis

**Figure 1:**
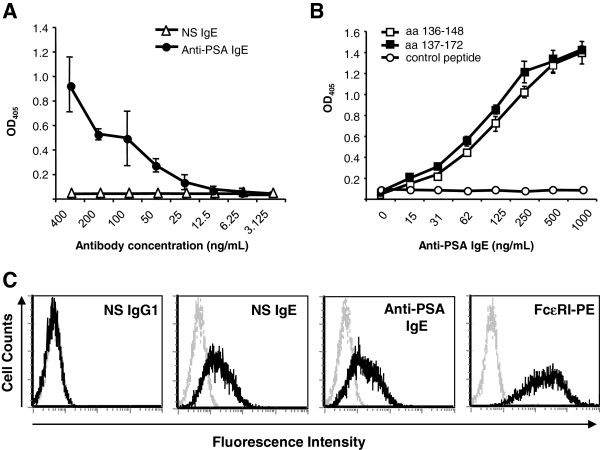
**Binding to antigen and FcεRIα. A**) Binding of the anti-PSA IgE compared to a NS IgE to PSA was detected by ELISA. **B**) Binding to the known epitope recognized by AR47.47 was confirmed by ELISA using PSA peptides containing the antigen binding site. Binding was detected by an AP-conjugated anti-human κ secondary antibody followed by a phosphatase substrate. ELISA data are the mean of triplicate samples with standard deviations indicated and are representative of 3 independent experiments. **C**) Binding to the FcεRIα on the surface of CHO-3D10 cells was detected using flow cytometry and an anti-human κ FITC-conjugated antibody. An anti-FcεRI PE-conjugated antibody was used simultaneously to verify receptor expression. Data are representative of 3 independent experiments.

Both anti-PSA antibodies (IgG1 and IgE) were properly assembled and secreted and showed the expected molecular weight of approximately 150 kDa and 190 kDa, respectively (Additional file 1: Figure S1) [18]. The anti-PSA IgE was shown by ELISA to bind the full-length PSA protein (Figure 1A), as well as the known epitope bound by AR47.47, which maps to the region of amino acids 137–144 of PSA (Figure 1B) [15]. These studies confirm that the AR47.47 variable regions are capable of binding antigen in the context of a human IgE molecule. Additionally, binding to the artificial HSA-PSA conjugate was also detected (data not shown). Furthermore, flow cytometry analysis showed binding of the IgE to human FcεRIα expressed on the surface of CHO-3D10 cells (Figure 1C). As expected similar levels of binding were observed for a non-PSA specific human IgE (NS IgE) indicating that the Fc region of the antibody is fully functional with respect to FcεRIα binding.

### IgE-induced degranulation of effector cells

**Figure 2:**
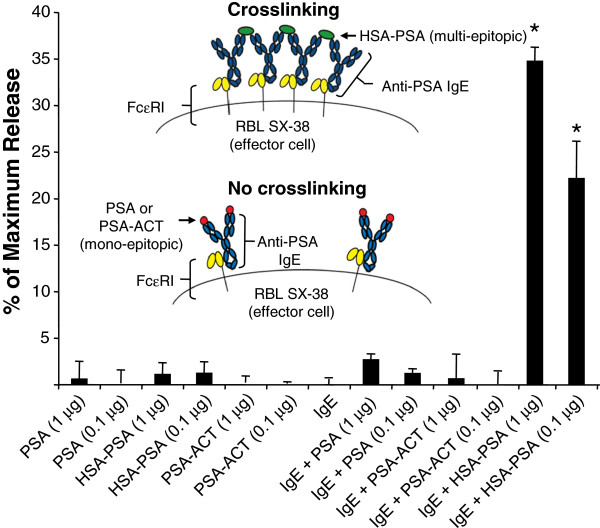
***In vitro *degranulation. **Rat basophil leukemic cells expressing human FcεRI (RBL SX-38) were sensitized with 1 μg of IgE or buffer alone for 2 hours, followed by a 2-hour incubation with PSA, PSA-ACT, or HSA-PSA (an artificial PSA multi-epitope conjugate). The antigen concentration was adjusted to the equivalent molar amount of PSA-ACT (1 µg/mL or 0.1 µg/mL). The release of β-hexosaminidase into cell supernatants was monitored through the addition of 2.5 μM *p*-nitrophenyl-*N*-acetyl-β-D-glucosamine in 50 mM citrate buffer (pH 4.5). The mean and standard deviation of triplicate samples are shown. Data are representative of 2 independent experiments * *p* < 0.01 (Student’s *t*-test) compared to either component alone.

In order to confirm that the human Fc region of the targeted IgE is functional, its ability to induce degranulation of FcεRI-bearing effector cells was evaluated. In the allergic reaction, mast cells and basophils coated with IgE rapidly degranulate in the presence of multi-epitopic antigens, due to cross-linking of the FcεRI and the subsequent Type I hypersensitivity/anaphylactic reaction [35] (Figure 2 inset). However, if a mono-epitopic antigen is present, the receptor is not expected to be cross-linked and degranulation should not occur. Therefore, we tested the two most abundant forms of PSA found in the blood of PCa patients (the full PSA protein and PSA-ACT), both of which interact with the anti-PSA IgE in a mono-epitopic manner. Additionally, we tested an artificial molecule (HSA-PSA) that consists of multiple PSA peptides containing the epitope bound by AR47.47 conjugated to human serum albumin (HSA) to mimic a multi-epitopic antigen. The anti-PSA IgE mediated degranulation of RBL SX-38 cells *in vitro* only when exposed to HSA-PSA (Figure 2), but not PSA or PSA-ACT alone. These data show that the Fc region of the antibody is functional and can induce degranulation of effector cells in the presence of a multi-epitopic antigen.

**Figure 3:**
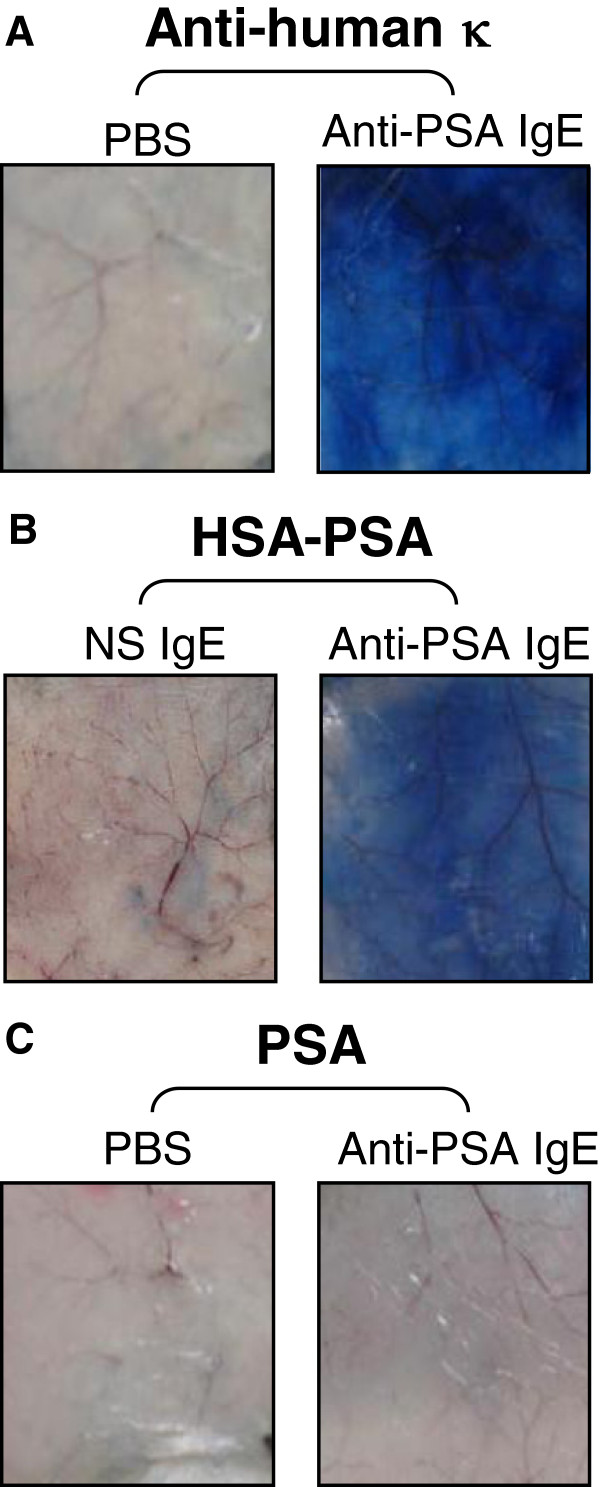
***In vivo *local, passive anaphylaxis in human FcεRIα transgenic mice. **Mice were injected intradermically on the back with PBS alone, 1μg of PSA IgE or a NS IgE. After 4 hours, mice were injected intravenously with either **A**) 25 μg anti-human κ, **B**) 50 μg of the HSA-PSA peptide (containing multiple epitopes for AR47.47) conjugate, or **C**) 50 μg PSA in 1% Evans blue in PBS. Animals were euthanized 10 minutes later. A local hypersensitivity/anaphylactic response (acute inflammatory reaction) caused by the degranulation of FcεRI-expressing cells in the skin is visualized by leakage of the dye into the skin due to vasodilation of local blood vessels. Representative images of 3 independent experiments are shown.

*In vivo* studies using human IgE can be hindered due to various limitations as discussed [22], most notably the lack of interaction of human IgE with murine FcεRI [20]. For this reason, BALB/c human FcεRIα transgenic mice were used to evaluate the ability of the anti-PSA IgE to induce degranulation of effector cells *in vivo*. In these animals, the FcεRI is a chimeric receptor that can be activated by human IgE with FcεRIα being of human origin while both the FcεRIγ and FcεRIβ chains are of murine origin [31-33]. A strong reaction was observed in the presence of the anti-PSA IgE when it was cross-linked using an anti-human κ antibody (Figure 3A) or the artificial multi-epitope form of the antigen, HSA-PSA (Figure 3B) indicating that the IgE is able to mediate an acute inflammatory (type I hypersensitivity) reaction *in vivo*. However, this inflammatory reaction was not observed in the presence of PSA (Figure 3C) or PSA-ACT (data not shown). Color intensity analysis assessed using the NIH ImageJ software demonstrated that the mean color intensity of 3 animals in the region where the anti-PSA IgE was injected was significantly higher in the presence of the anti-human κ antibody or HSA-PSA (*p* < 0.01, Student’s *t* test). The mean color intensity did not increase in the presence of PSA or PSA-ACT (data not shown). These studies are consistent with our *in vitro* degranulation studies and suggest that the anti-PSA IgE would not cause a systemic hypersensitivity (anaphylactic) reaction in the presence of the most abundant forms of PSA in blood (free or complexed to ACT).

### IgE-facilitated antigen stimulation

**Figure 4:**
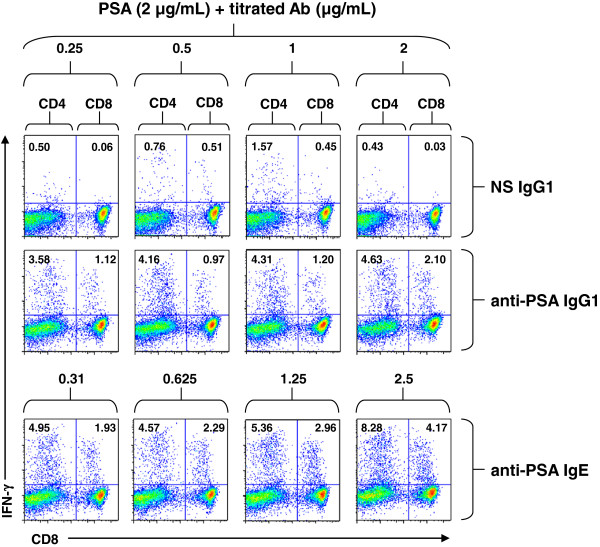
***In vitro *antigen stimulation assay. **DC were generated from healthy donor monocytes using GM-CSF and IL-4. On Day 7, DC were loaded with 2 μg/mL PSA and various concentrations of either a NS IgG1 or the anti-PSA IgG1. DC were also loaded with equimolar amounts of the anti-PSA IgE antibody. Four hours after loading, the DC were matured with TNF-α and IFN-α. Autologous T cells were added 24 hours later. Cells were incubated for 7 days and restimulated with freshly generated and pulsed DC weekly. The cells were stimulated for a total of 3 rounds. One day after the third stimulation in the presence of Brefeldin A, T cells were harvested and analyzed for activation of CD4 and CD8 IFN-γ producing T cells by intracellular cytokine staining and flow cytometry. CD3^+^/CD8^-^ (CD4, left quadrants) and CD3^+^/CD8^+ ^(CD8, right quadrants) that stain positively for IFN-γ are a measure of specific activation.

DC are potent APC that are capable of processing antigens for presentation in the context of the major histocompatibility complexes I and II (MHC I and II) [36,37]. Thus, DC are capable of processing antigens via cross-presentation leading to the activation of a cell-mediated immune response. Human DC express both FcεRI and FcεRII [20,21] and are able to interact with human IgE bound to antigen [38-42]. IgE-facilitated antigen presentation has been shown to occur through the binding of human IgE to either FcεRI [39] or CD23 [42]. Processing and presentation has also been shown to be required for the activation of T cells through IgE-facilitated antigen presentation [42]. Thus, we evaluated the ability of the anti-PSA IgE to induce antigen presentation and T-cell activation *in vitro* in human DC obtained from healthy individuals. DC pulsed with the IgE complexed with PSA induced CD4 and CD8 T-cell activation (Figure 4). Importantly, the activation of CD8 cells is indicative of cross-presentation. DC pulsed with the anti-PSA IgG1 complexed to PSA also induced CD4 and CD8 T-cell activation, although to a lesser extent (Figure 4). Studies conducted with cells isolated from 2 additional donors also showed increased T-cell activation in the presence of DC loaded with the anti-PSA IgE complexed with PSA. Taken together, these data suggest that the anti-PSA IgE is capable of enhancing antigen presentation *in vitro*.

### Vaccination study

**Figure 5:**
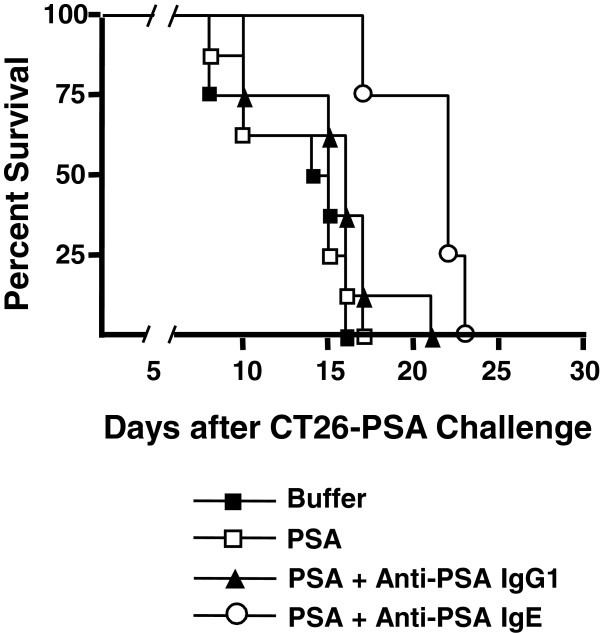
**Vaccination study in human FcεRIα transgenic mice. **Survival plot of human FcεRIα transgenic mice vaccinated subcutaneously in the left flank with PSA alone (4 μg) or complexed in a 1:1 molar ration to either the anti-PSA IgE or IgG1. Mice were given a booster on day 15, also in the left flank. Two-weeks after the booster on day 40, mice were challenged with 10^6 ^murine CT26 cells expressing human PSA (CT26-PSA) subcutaneously in the right flank. Animals admininstered with buffer alone were used as negative controls. The survival plot shown is the combined data from 2 independent experiments with a total of 8 mice per group and was generated in GraphPad Prism.

In order to evaluate the ability of the anti-PSA IgE to elicit an immune response that would lead to anti-cancer activity *in vivo*, a vaccination strategy was employed. Sera collected from the animals at day 14 and day 28 were examined for a murine anti-human PSA immune response. Titers of both murine IgG1 (indicative of a T_H_2/humoral response) and IgG2a (indicative of a T_H_1/cellular response [43,44]) specific for PSA were determined. Vaccination with human PSA alone resulted in the induction of an IgG1 response in 5 out of 8 animals (Table 1). Following vaccination with complexes of PSA and the anti-PSA IgG1 or the anti-PSA IgE, all 8 animals developed a murine IgG1 response (Table 1). After the initial immunization, only IgG1 titers were significantly higher in animals vaccinated with complexes of the anti-PSA IgG1 and PSA (*p* = 0.041 with a titer greater than 900, Fisher Exact test) compared to animals immunized with PSA alone. However, after the booster, IgG1 levels were no longer significantly different. Murine IgG2a responses to PSA were induced in 5 out of 8 animals vaccinated with PSA alone (Table 2). Immunization with PSA complexed to the anti-PSA IgG1 results in the same number of IgG2a positive animals, however, titers were low (Table 2). An IgG2a response was observed in 7 out of 8 animals vaccinated with PSA complexed to the anti-PSA IgE (Table 2). After the booster, significantly higher titers of murine IgG2a were observed in these mice (Table 2). These titers were significantly higher compared to complexes of PSA and the anti-PSA IgG1 (*p* = 0.001 with a titer greater than of 50) or compared to PSA alone (*p* = 0.041 with a titer greater than 50, Fisher Exact test). An unusually high titer of IgG2a was observed in 1 animal after the booster with PSA alone. Interestingly, vaccination with the anti-PSA IgE complexed to PSA significantly prolonged survival of mice subcutaneously challenged two-weeks after the booster (on day 40) with CT26-PSA cells (Figure 5, Table 3). However, this protection was not observed with vaccination with the IgG1 complexed to PSA or with PSA alone (Figure 5; Table 3).

**Table 1:** Murine anti-human PSA IgG1 titers in the serum of vaccinated human FcεRIα transgenic mice

**Titers 14 days after initial immunization**				
Mouse #	PSA + anti-PSA IgE	PSA + anti-PSA IgG1	PSA	Buffer
1	8,100	100	900	0
2	2,700	24,300	0	0
3	900	8,100	0	0
4	2,700	24,300	0	0
5	2,700	24,300	2,700	0
6	900	72,900	8,100	0
7	24,300	8,100	900	0
8	900	2,700	900	0
Median	2,700	16,200	900	0
**Titers 13 days after booster (28 days after initial immunization)**				
Mouse #	PSA + anti-PSA IgE	PSA + anti-PSA IgG1	PSA	Buffer
1	218,700	8,100	24,300	0
2	218,700	72,900	0	0
3	72,900	24,300	0	0
4	218,700	218,700	0	0
5	72,900	400,000	218,700	0
6	72,900	218,700	218,700	0
7	24,300	218,700	8,100	0
8	218,700	24,300	72,900	0
Median	145,800	145,800	16,200	0

Data shown are combined from two independent experiments (n = 4) with a total of 8 animals per group. Sera were serially diluted 3-fold starting at 1:100. Titer was determined as the highest dilution from which a positive signal was obtained.

**Table 2:** Murine anti-human PSA IgG2a titers in the serum of vaccinated human FcεRIα transgenic mice

**Titers 14 days after initial immunization**				
Mouse #	PSA + anti-PSA IgE	PSA + anti-PSA IgG1	PSA	Buffer
1	0	0	0	0
2	0	0	0	0
3	200	0	0	0
4	0	0	0	0
5	0	0	50	0
6	0	0	0	0
7	0	0	0	0
8	100	0	0	0
Median	0	0	0	0
**Titers 13 days after booster (28 days after initial immunization)**				
Mouse #	PSA + anti-PSA IgE	PSA + anti-PSA IgG1	PSA	Buffer
1	0	0	1,600	0
2	200	0	50	0
3	200	50	0	0
4	800	0	0	0
5	200	50	400	0
6	400	50	0	0
7	100	50	50	0
8	100	50	50	0
Median	200	50	50	0

Data shown are combined from two separate experiments (n = 4) with a total of 8 animals per group. Sera were serially diluted 2-fold starting at 1:50. Titer was determined as the highest dilution from which a positive signal was obtained.

**Table 3:** Median survival of animals in the vaccination study in human FcεRIα transgenic mice

	**Median survival (days)**	***p*-value (compared to buffer control)**	***p*-value (compared to PSA)**	***p*-value (compared to PSA + anti-PSA IgG1)**
Buffer	14.5			
PSA	15	0.777		
PSA + anti-PSA IgG1	16	0.086	0.147	
PSA + anti-PSA IgE	22	0.004	0.005	0.01

Data shown are combined from two independent experiments (n = 4 for each) with a total of 8 animals per group.

## Discussion

In general, most therapeutic antibodies used in the clinic to treat malignancies are of the IgG class [16,17]. However, the IgE class of antibodies may also be a meaningful option for this purpose. In fact, a tumor targeted murine IgE specific for either the major envelope glycoprotein (gp36) of mouse mammary tumor virus (MMTV) [45] or an antigenic determinant on the surface of human colon carcinoma cells [46] have shown anti-cancer effects in murine models. Additionally, a mouse/human chimeric IgE (MOv18 IgE) specific for the human ovarian cancer antigen folate binding protein (FBP) demonstrated superior anti-tumor activity in murine models compared to a mouse/human chimeric IgG1 with the same variable regions (MOv18 IgG1) [47,48]. This anti-cancer activity was only observed in the presence of human PBMC or human monocytes, demonstrating that the Fc effector functions of the antibody are required for the observed inhibitory activity [47-49]. Moreover, the MOv18 IgE was shown to mediate both antibody-dependent cell-mediated cytotoxicity (ADCC) and antibody-dependent cell-mediated phagocytosis (ADCP) *in vitro* through the interaction of FcεRI and CD23, respectively [50]. A humanized anti-HER2/*neu* IgE with the variable regions of trastuzumab (Herceptin^®^) has also been shown to trigger basophil degranulation and ADCC *in vitro* in the presence of human HER2/*neu* expressing cells [51]. More recently, a fully human IgE also targeting human HER2/*neu* with the variable regions of the scFv C6MH3-B1 has been described and shown to have *in vivo* anti-cancer activity in human FcεRIα transgenic mice in a passive immunotherapeutic setting against a syngeneic tumor expressing human HER2/*neu*[25]. Complexes of this IgE with its soluble antigen (ECD^HER2^) demonstrated enhanced *in vitro* antigen presentation when compared to an anti-HER2/*neu* IgG1 complexed to ECD^HER2^. This study also described a preliminary toxicological study carried out in *Macaca fascicularis* (cynomolgus) monkeys and showed that this anti-HER2/*neu* IgE was well-tolerated in this initial study at the 2 doses tested [25].

We now report the development of a mouse/human chimeric IgE specific for PSA that contains the variable regions of the murine antibody AR47.47 and binds the PSA antigen and FcεRI. Cross-linking induced by either the artificial HSA-PSA conjugate that contains multiple copies of the AR47.47 epitope or the anti-human κ antibody showed that this IgE is capable of inducing degranulation of effector cells both *in vitro* and *in vivo*. However, degranulation of effector cells sensitized with the anti-PSA IgE did not occur in the presence of the natural PSA protein or the PSA-ACT complex found in the blood which carry a single binding epitope for AR47.47 per PSA molecule. We used a PSA concentration in our *in vivo* studies that exceeds the amount of PSA found in human blood: we injected 50 μg of protein per animal, which is equivalent to 25–50 μg/mL, given the blood volume of a 18–25 gram mouse is 1–2 mL [52]. The median total PSA level in the blood of normal human males varies with age but ranges from 0.6-1.5 ng/mL [9,53]. Total PSA levels in the blood of prostate cancer patients are usually much higher. However, these levels are also highly variable (ranging from 10–1000 ng/mL) [9]. Thus, even at the initial high concentration in blood used for our studies, no reaction was observed. Our data suggest that physiological or pathological levels of PSA or PSA-ACT in the blood are not likely to induce a systemic anaphylactic reaction if combined with the anti-PSA IgE. It is important to note that PSA can also be found in the blood of cancer patients complexed with A2M and API, although these complexes are present at very low levels [9-12]. Complexes of PSA with API [54] have been shown to occur at a 1:1 ratio and, therefore, should not induce a systemic reaction. Tetrameric A2M can bind two PSA molecules; however, the A2M-PSA complex cannot be detected in the blood by immunoassays since PSA is encapsulated by A2M [55,56]. Therefore, it is expected that the anti-PSA IgE would not be able to bind the encapsulated PSA in this complex and thus, it would not be expected to trigger a systemic anaphylactic reaction. Further studies are needed to determine if these complexes can induce degranulation of effector cells in the presence of the anti-PSA IgE as well as the safety profile of this antibody.

Although the interaction with the anti-PSA IgE and naturally occurring soluble forms of the antigen did not induce degranulation of effector cells, immune complexes of the IgE with the soluble antigen may interact with APC. DC are potent APC that are capable of cross presentation. DC are able to interact with human IgE bound to antigen [38-42], including complexes consisting of mono-epitopic interactions [57]. Both FcεRI [39] and CD23 [42] can mediate IgE-facilitated antigen presentation. Previous studies suggest that PSA is an appropriate target to mobilize T-cell mediated immunity. PCa patients have been found to contain naturally occurring IFN-γ producing CD8^+^ T-cells that respond to PSA peptide in primary cultures [58]. Additionally, a PSA DNA vaccine induced a robust PSA protein and peptide specific T-cell response in 5 of 6 patients [59]. Furthermore, IgE has been shown to act as an adjuvant for cancer therapy [24], making the anti-PSA IgE a meaningful strategy to explore for the induction of a cell-mediated immune response in PCa patients. In the present studies, enhanced *in vitro* activation of both CD4 and CD8 T cells was observed when these cells were exposed to DC loaded with the anti-PSA antibodies complexed to PSA. However, cytotoxic T-cell (CTL) studies were not conducted. These data are consistent with the enhanced presentation previously observed with complexes of the parental murine anti-PSA antibody AR47.47 and PSA [15] and with the fact that a T-cell response can be elicited *in vitro* in an antigen stimulation assay using PBMC isolated from normal donors [60]. However, T-cell activation was higher with IgE immune complexes than what was observed with immune complexes containing the mouse/human chimeric anti-PSA IgG1. Enhanced antigen presentation with complexes of an anti-HER2/*neu* IgE and the soluble antigen, compared to complexes of the antigen and an anti-HER2/*neu* IgG1 has also been previously observed in human DC *in vitro*[25]. Importantly, the activation and expansion of CD8 cells, indicative of cross-presentation, was only observed in either case when DC were loaded with complexes of the IgE or IgG1 with PSA. Complexes of IgE and PSA were particularly efficient in cross-presentation (CD8 T-cell activation). Taken together, these results support the hypothesis that antigen-specfic IgE can enhance antigen-specific T-cell immunity. However, further studies are needed to understand this property and compare its ability to that of IgG1.

The *in vivo* vaccination studies show prolongation of survival in mice vaccinated with complexes of the anti-PSA IgE and PSA as compared to buffer, PSA alone, or anti-PSA IgG1 complexed with PSA. The effect, under these initial conditions, is modest but significant, and the data show a survival advantage with the vaccination strategy utilizing complexes of IgE and its targeted antigen. Evaluation of the murine anti-PSA response showed that murine IgG1 levels are increased in all experimental groups. It is not surprising that PSA alone induced a relevant IgG1 response since PSA is a human protein. However, the titers of IgG1 in mice vaccinated with either the IgE or the IgG1 complexed to the antigen were increased compared to PSA alone, consistent with a stronger induction of a T_H_2 (humoral) immune response. Additionally, in animals vaccinated with complexes of the anti-PSA IgE and PSA, significantly higher titers of murine IgG2a response were also observed. This suggests that this vaccination strategy also induced a T-cell mediated response (T_H_1) in these animals. Since this was the only group to show significant protection from tumor challenge, these findings may correlate with a preferential induction of a T_H_1 response by vaccination with the complexes of the anti-PSA IgE and the PSA antigen. Even though the *in vivo* effects observed with the anti-PSA IgE were modest, the observed anti-cancer effect may have clinical significance, since the BALB/c strain used in these studies is biased towards a T_H_2 response [61,62]. However, it is important to note that in our model system, the constant regions of the anti-PSA IgE and IgG1 are of human origin (xenotypic in the mouse) and that this might influence the immunogenicity of PSA. Additional studies are needed to further understand the observed immunoactivation and anti-cancer activity in this model as well as in other models.

Complexes of PSA with the anti-PSA IgG1 did not show an anti-cancer effect in our particular vaccination protocol. However, increased *in vitro* T-cell activation was observed, even though it was to a lesser extent compared to complexes of PSA with the IgE. The enhanced T-cell activation by the anti-PSA IgG1 observed in this study is consistent with a previous study that showed increased PSA-specific T-cell activation in a vaccination setting that used complexes of PSA and the anti-PSA IgG1 in human PSA transgenic mice [63]. This study demonstrated that the anti-PSA IgG1 is able to break tolerance and suggests that in a therapeutic setting this response can be further enhanced. Taken together, the above data suggest that the anti-PSA IgE might also have similar effects under tolerogenic conditions, a hypothesis that could not be evaluated in the human PSA transgenic animal due to the lack of expression of human FcεRIα.

It is tempting to speculate that the anti-PSA IgE can also target PCa tumors and elict an acute inflammatory reaction and thereby, enhance anti-tumor activity. Even though PSA is a secreted antigen, it might be concentrated within the tumor microenvironment. For instance, this may occur due to interactions with glycosaminoglycans on the surface of malignant cells or in the tumor stroma through its heparin-binding activity. PSA has two proposed heparin-binding sites [64] and has been shown to bind heparin [65,66]. Previous studies have shown that anti-PSA antibodies can have anti-cancer activity *in vitro* and *in vivo*[13,14], suggesting that such antibodies are able to target PCa tumors. In addition, a radiolabeled anti-PSA monoclonal antibody was used target PCa tumors in human for imaging purposes [67]. Thus, it is possible that the IgE antibody may also have this activity. Further studies are needed to evaluate this possibility.

## Conclusions

The studies described here belong to the field of AllergoOncology, which aims to evaluate IgE-mediated anti-tumor immune responses with the goals of furthering our understanding of the biology of IgE and developing IgE-based cancer therapies. In this article, we describe the development of a novel antibody of the IgE class that expands this field and supports the notion that this anti-PSA IgE has potential therapeutic value in humans. However, additional evaluation of the anti-PSA IgE is warranted to further understand both the anti-cancer effects and the safety profile of the molecule.

## Competing interests

CFN and BCS are advisors to and own shares in Quest PharmaTech, Inc. (Edmonton, Alberta, Canada). All other authors have no competing interests to disclose.

## Authors’ contributions

TRDW performed cloning and expression of the antibodies and was principally responsible for conducting the binding analysis and the *in vivo* studies; participated in the study design, performed the statistical analysis, and drafted the manuscript. GH participated in the construction and expression of the antibodies, worked on the *in vivo* local anaphylaxis assay, and participated in the study design. RKL carried out the *in vitro* degranulation assays and participated in the study design. RQ participated in the *in vitro* degranulation assays and in all *in vivo* studies. MK participated in ELISA studies of the recombinant antibodies and murine sera. JAR and EOS participated in the production of the antibodies and in the study design. OMM participated in the study design, helped with study coordination, and contributed to manuscript preparation. BCS and CFN supervised the project, designed and performed peptide conjugations, selected the antigen binding epitope, and conducted the *in vitro* antigen stimulation assays. MLP supervised the project, participated in study design and coordination, and helped to prepare the manuscript. All authors read and approved the final manuscript.

## Pre-publication history

The pre-publication history for this paper can be accessed here:

http://www.biomedcentral.com/1471-2407/13/195/prepub

## Supplementary Material

Additional file 1: Figure S1 Cloning strategy for production of the mouse/human chimeric anti-PSA antibodies. The DNA encoding both the light chain and heavy chain variable regions was obtained from hybridoma cells expressing the murine anti-human PSA antibody AR47.47 and cloned into pCR-BluntII-TOPO vectors. The DNA encoding the variable regions was then subcloned into the respective expression vectors. To create the anti-PSA IgG1, both the light chain κ and heavy chain γ1 expression vectors were electroporated into the murine myeloma cell line Sp2/0-Ag14 . The DNA encoding the heavy chain PSA variable region was further subcloned into the IgE heavy chain expression vector and electroporated (together with the PSA κ expression vector) into Sp2/0-Ag14 cells to produce the IgE antibody. Both chimeric antibodies were properly assembled and secreted. The purified antibodies show the expected molecular weight as demonstrated by SDS-PAGE under both non-reducing and reducing conditions. (PPT 1384 kb) Click here for file
