# Supplementary figures and images for: A novel IgE antibody targeting the prostate-specific antigen as a potential prostate cancer therapy
Source: BMC Cancer. 2013 Apr 17;13:195. doi: 10.1186/1471-2407-13-195 (PMC3651304; doi:10.1186/1471-2407-13-195)

## Slide 1
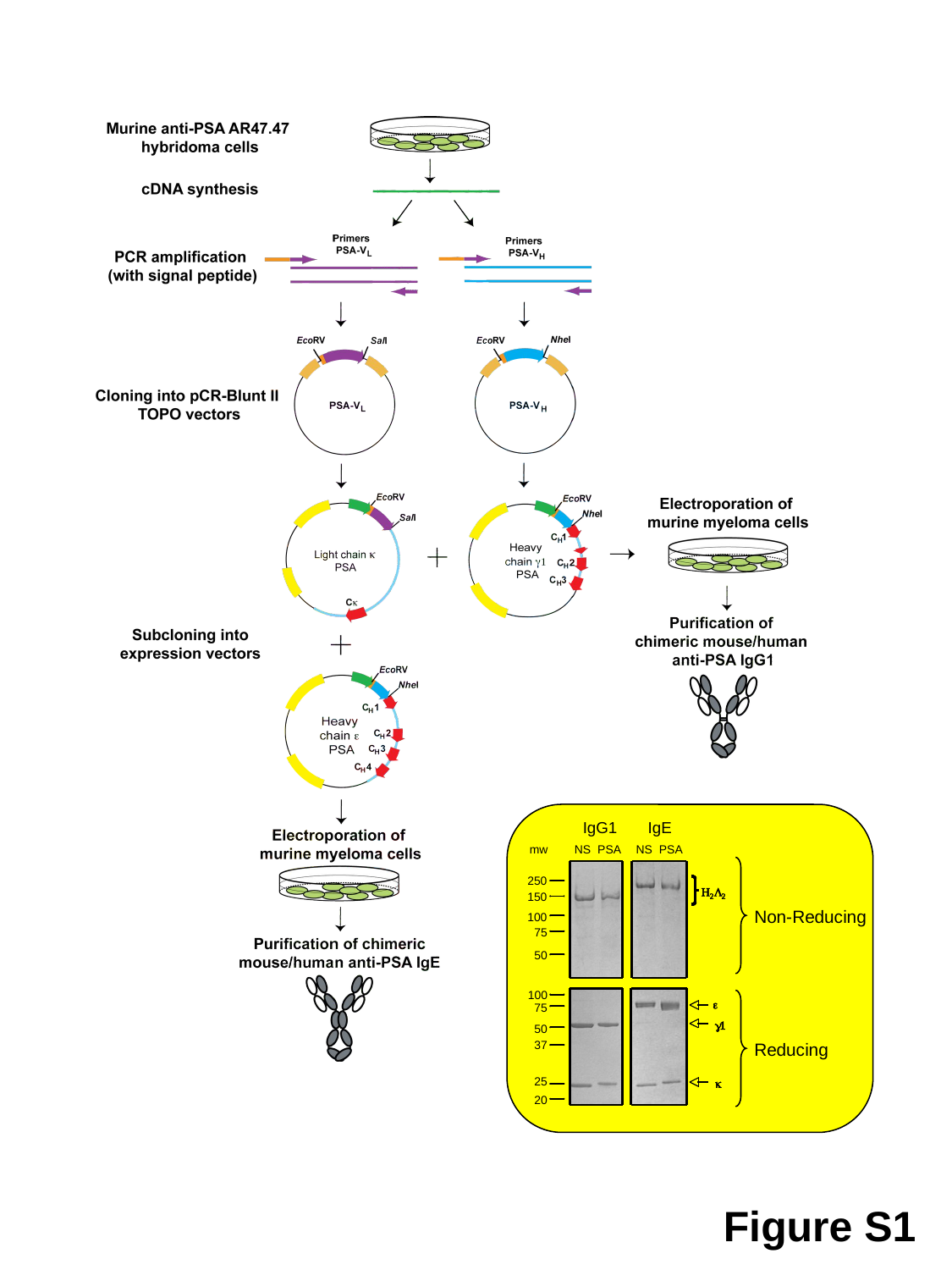

Non-Reducing
Reducing
Figure S1

Supplement: Additional file 1: Figure S1 — Cloning strategy for production of the mouse/human chimeric anti-PSA antibodies. The DNA encoding both the light chain and heavy chain variable regions was obtained from hybridoma cells expressing the murine anti-human PSA antibody AR47.47 and cloned into pCR-BluntII-TOPO vectors. The DNA encoding the variable regions was then subcloned into the respective expression vectors. To create the anti-PSA IgG1, both the light chain κ and heavy chain γ1 expression vectors were electroporated into the murine myeloma cell line Sp2/0-Ag14 . The DNA encoding the heavy chain PSA variable region was further subcloned into the IgE heavy chain expression vector and electroporated (together with the PSA κ expression vector) into Sp2/0-Ag14 cells to produce the IgE antibody. Both chimeric antibodies were properly assembled and secreted. The purified antibodies show the expected molecular weight as demonstrated by SDS-PAGE under both non-reducing and reducing conditions. (PPT 1384 kb) [file 1471-2407-13-195-S1.ppt]
